# Comparative effectiveness of different surgical timings on neurological outcomes for cranioplasty: Protocol for a prospective non-randomized controlled trial

**DOI:** 10.1371/journal.pone.0318841

**Published:** 2025-03-10

**Authors:** Jingguo Yang, Xingyu Zhang, Xiaoyu Yang, Junjie Wang, Chao You, Lu Ma, Junwen Guan

**Affiliations:** 1 Department of Neurosurgery, West China Hospital, Sichuan University, Chengdu, Sichuan Province, P.R. China; 2 Department of Communication Science and Disorders, School of Health and Rehabilitation Sciences, University of Pittsburgh, Pittsburgh, Pennsylvania, United States of America; Universiti Sains Malaysia, MALAYSIA

## Abstract

**Background:**

Cranioplasty (CP), a surgical procedure that restores cranial integrity and potentially enhances neurological outcomes, is commonly performed following decompressive craniectomy for various reasons. However, there is considerable controversy and variation regarding the optimal timing for cranioplasty, particularly concerning its impact on neurological functional outcomes. This paper outlines the protocol for a multicenter, non-randomized controlled trial designed to investigate whether the timing of cranioplasty influences neurological outcomes.

**Methods/Design:**

This study will be conducted from June 2025 to June 2026 across multiple clinical centers in China, targeting the enrollment of at least 500 adults aged 18-65 years with skull defects larger than 25 cm². Participants will be divided based on the timing of their cranioplasty relative to decompressive craniectomy into two groups: early (within 3 months post-decompression) and late (after 3 months). The primary outcome, assessed through the Barthel Index, will measure functional recovery 6 months post-surgery, with secondary outcomes including mortality, quality of life, cognitive performance and complication rates.

**Discussion:**

This non-randomized clinical trial focuses on the neurological outcomes associated with different timings of cranioplasty. It is anticipated that the findings will contribute valuable insights and support more informed clinical decisions regarding the timing of cranioplasty. By comparing early and late cranioplasty, the trial aims to clarify how timing affects recovery and overall neurological improvement post-surgery.

**Trial Registration:** ChiCTR2400094619

## 1. Introduction

In patients with severe traumatic brain injury (TBI), malignant ischemic stroke, or intracranial hemorrhage, intracranial pressure can be elevated due to hematomas, contusions, or brain swelling. Decompressive craniectomy (DC) is a surgical procedure aimed at reducing intracranial pressure and lowering mortality rates [1,2]. Cranial reconstruction, usually undertaken weeks to months later, offers both a protective barrier and cosmetic reconstruction [3,4]. Additionally, studies indicate that cranioplasty normalizes cerebrospinal fluids (CSF) and vascular circulation, while also enhancing neurological functional outcomes [5–8].

Although the surgical technique for cranioplasty is relatively simple, it is associated with high rates of complications and poor outcomes [9–12]. The interval between decompressive craniectomy and cranioplasty has received considerable attention because timing is known as a potentially modifiable factor [13–18]. The timing of cranioplasty varies substantially between centers. A recent consensus statement proposed defining a threshold for early versus late cranioplasty as useful for research purposes, with early cranioplasty defined as 6 weeks to 3 months post-craniectomy, and intermediate to delayed cranioplasty defined as occurring later than 3 months [16]. Existing evidence presents conflicting views on whether the timing of cranioplasty affects complication rates and neurological function outcomes [14,19–21]. Although a recent comprehensive systematic review concluded that early cranioplasty could enhance neurological function, the strength of the supporting evidence is relatively low, consisting only of retrospective and small-scale studies [7]. Therefore, the relationship between the timing of cranioplasty and its outcomes remains controversial.

Given the ongoing controversy and the potential for modifiable outcomes through timing adjustments, this study aims to rigorously evaluate the impact of different cranioplasty timings on neurological function and complication rates in a structured, non-randomized controlled trial format. By comparing groups of patients who receive early versus late cranioplasty following DC, this trial seeks to provide clearer insights into optimal timing for cranioplasty, thus informing future clinical practices. The study is designed to fill the gap in high-quality, prospective data concerning the timing of this crucial intervention.

## 2. Methods and analysis

### 2.1. Study objectives

The primary objective of this non-randomized controlled trial is to determine the impact of early versus late cranioplasty on neurological outcomes in patients following decompressive craniectomy. Specifically, the study aims to measure and compare functional neurological outcomes, primarily using standardized indices like the Barthel Index, between patients who undergo cranioplasty within 6 weeks to 3 months post-decompression (termed early cranioplasty) and those who receive the procedure later than 3 months post-decompression (termed late cranioplasty).

In addition to the primary objective, the study has several secondary objectives. One key secondary objective is to compare the rates of surgical complications between the early and late cranioplasty groups. This includes assessing the frequency and severity of complications such as infections, seizures, or mechanical issues with the cranioplasty material, such as displacement or rejection. Another important secondary goal is to evaluate other functional outcomes and quality of life measures, including cognitive performance (e.g., using the Mini-Mental State Examination), mobility, and patient-reported satisfaction or quality of life changes post-surgery. Furthermore, the study will investigate any differences in the length of hospital stay and subsequent care requirements between the two groups, which could have implications for healthcare resource utilization and overall treatment cost-effectiveness.

By addressing these objectives, the study seeks to provide comprehensive data on how the timing of cranioplasty influences not only the direct neurological outcomes but also the broader implications for patient recovery and healthcare system burdens. The results could potentially lead to refined guidelines for the timing of cranioplasty to optimize patient outcomes post-decompressive craniectomy.

### 2.2. Study design

This study is designed as a prospective, multicenter, non-randomized controlled trial to evaluate the outcomes of early versus late cranioplasty in patients following decompressive craniectomy. The trial is organized to compare two distinct patient groups based on the timing of their cranioplasty procedure. The early group will consist of patients receiving cranioplasty within 6 weeks to 3 months post-decompression, while the late group will include those undergoing the procedure later than 3 months post-decompression. The trial will enroll participants from 10 clinical centers across China, selected based on their high standards of care and previous experience with similar studies. This diverse set of locations and inclusion of patients with varied primary pathologies—such as severe traumatic brain injury, malignant ischemic stroke, hemorrhagic stroke, and infiltrative tumors—ensures a broad patient demographic and enhances the generalizability of the study findings.The timing of cranioplasty for each participant will be determined based on clinical judgment, patient preference, and logistical factors, while still facilitating a controlled comparison of outcomes.

The study involving human participants has been reviewed and approved by Biomedical Research Ethics Committee of West China Hospital, Sichuan University (NO. 2024-1992). Also, the study has been registered at the Chinese Clinical Trial Registry (ChiCTR2400094619. Date: December 25, 2024.).

**Fig 1:**
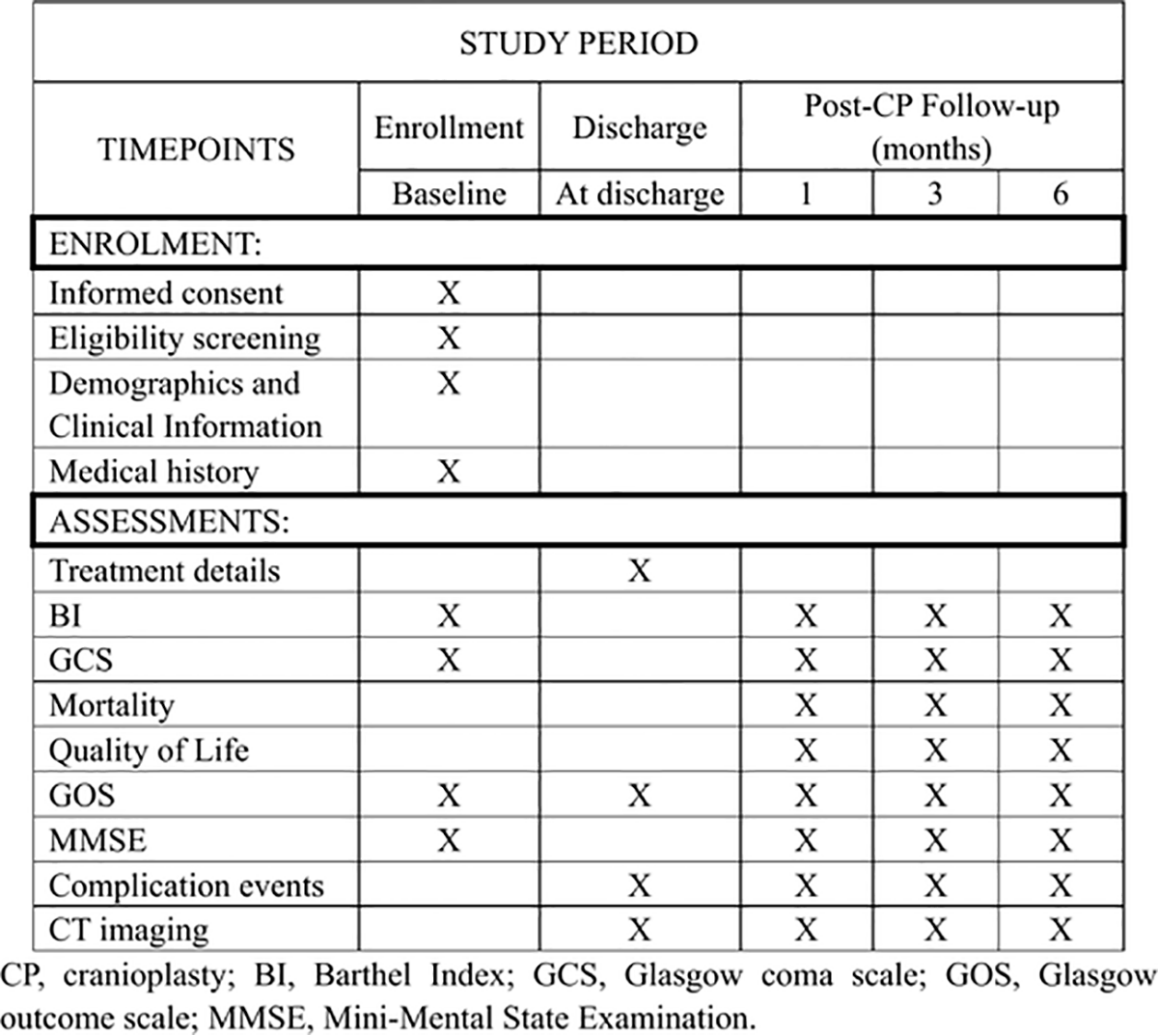
SPIRIT schedule of patients’ enrollment and assessments.

**Fig 2:**
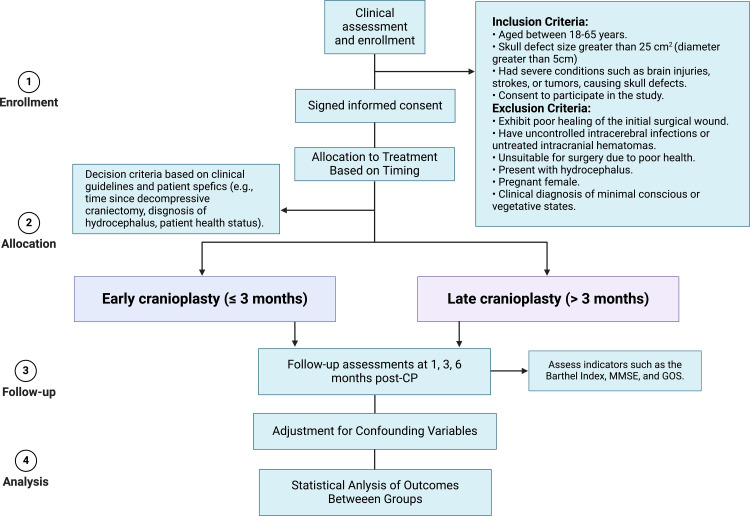
Flow chart of the study.

A total of at least 500 patients who have undergone decompressive craniectomy are expected to be included from June 2025 to June 2026. Each participating center was evaluated through a structured, multi-domain questionnaire to verify its suitability for this study. This evaluation focused on each center’s historical experience with cranioplasty cases, capacity for high patient volume, quality of postoperative outcomes, and availability of necessary surgical resources. Centers with a record of extensive cranioplasty experience were prioritized, ensuring that each site is fully capable of managing these cases effectively. The qualifications and experience of the surgeons were also carefully reviewed. Each participating surgeon is required to have at least ten years of clinical experience in neurosurgery and a record of performing cranioplasty procedures. These criteria ensure that only highly experienced and board-certified neurosurgeons will be conducting the procedures. Some centers also provided details about their surgeons’ prior involvement in relevant professional development activities and previous participation in randomized controlled trials, which reinforces their familiarity with clinical research standards[22,23]. To ensure consistency in data collection and outcome assessment, research personnel from all centers will undergo centralized training. This training will standardize the procedures for measuring neurological function outcomes and other relevant variables, ensuring that data are collected uniformly across all sites. Each participant will be followed for a minimum period of 6 months, with scheduled assessments to monitor their recovery and any complications. The organization and timing of data collection is outlined in Fig 1. The flow chart of this stufy is provided in Fig 2.

### 2.3. Study population

Recruitment for this study is scheduled to commence in June 2025 and is anticipated to conclude by June 2026. We aim to enroll a minimum of 500 patients who have undergone decompressive craniectomy. Patients will be allocated into two groups based on the timing of their cranioplasty: approximately 250 patients will undergo early cranioplasty (within 3 months post-decompression), and around 250 patients will receive late cranioplasty. The study will run until December 2026, which marks the final follow-up assessments for the last recruited participants.

The recruitment process will involve several strategies:

1. **Outpatient Evaluation**: Potential participants will initially undergo a comprehensive outpatient evaluation to assess their eligibility based on the study’s criteria.
2. **Medical Record Review**: Researchers will review the medical records of patients who have previously undergone decompressive craniectomy to identify candidates for subsequent cranioplasty.
3. **Direct Recruitment**: Senior neurosurgeons will actively recruit consecutive patients who meet the study’s inclusion and exclusion criteria during their clinical appointments.

#### 2.3.1. Inclusion criteria.

Participants eligible for this study must meet the following criteria:

- Aged between 18 and 65 years.
- Have a skull defect size greater than 25 cm² (diameter greater than 5 cm).
- Have undergone a previous decompressive craniotomy.
- The skull defect must have resulted from one of the following conditions, which will be recorded for each patient: severe traumatic brain injury, malignant ischemic stroke, hemorrhagic stroke, or an infiltrative tumor.
- Provide informed consent to participate in the study, either personally or through a legally authorized representative.

#### 2.3.2. Exclusion criteria.

Patients will be excluded from participating in the study if they:

- Exhibit poor healing from the initial decompressive craniotomy surgeries.
- Have uncontrolled intracerebral infections, defined as an infection that persists despite standard antibiotic treatment.
- Have untreated intracranial hematoma.
- Have a history of cranial osteromyelitis.
- Present with hydrocephalus.
- Have a clinical diagnosis of being in a minimally conscious state or a vegetative state.
- Are deemed unsuitable for surgery due to poor general health conditions.
- Are pregnant at the time of recruitment.

### 2.4. Treatment strategies

#### 2.4.1. *Timing of cranioplasty .*

In accordance with the international consensus on post-traumatic CP, we have set 3 months post-decompressive craniectomy as the threshold to distinguish early from late cranioplasty [16]. Patients will be categorized into two groups based on the timing of their cranioplasty: early cranioplasty (within 3 months of decompressive craniectomy) and late cranioplasty (beyond 3 months). To ensure a clear separation between the groups, we have implemented a two-week buffer window around the 90-day threshold. As a result, surgeries for the early group will occur no later than day 76 post-craniectomy, and surgeries for the late group will begin no earlier than day 104. This window minimizes overlap and enhances the distinction between the two timing groups. While the exact scheduling of cranioplasty may be influenced by factors such as surgeon availability and hospital scheduling, every effort will be made to adhere to these timing guidelines and to schedule the procedure within one week of patients reaching their designated timing group. Any deviations from this planned timing will be documented along with the reasons, to assess the impact of scheduling variability on study outcomes.

#### 2.4.2. *Surgical procedure .*

The choice of cranioplasty materials includes autologous bone and synthetic materials such as Polymethylmethacrylate (PMMA), Titanium, Polyetheretherketone (PEEK), and Hydroxyapatite (HA). Based on responses from our center enrollment questionnaire, the materials selected for use in this study are Titanium and PEEK, due to their widespread acceptance and use across the participating centers.

The steps involved in the cranioplasty procedure are as follows:

**1. Pre-operative Preparation:**

- Once a patient is included in the study, cranial spiral CT imaging (slice thickness ≤ 1.0 mm) will be performed to acquire detailed cranial data and aid in the generation of a virtual 3D model of the skull.
- Customization of the Titanium or PEEK plate based on the 3D model will ensure a patient-specific fit.

**2. Surgical Execution:**

- Patients will undergo routine pre-operative laboratory tests and hair removal one day prior to the procedure.
- The operation will commence with careful skin disinfection, followed by incisions along the original decompressive surgery lines.
- Scalp and temporal muscle will be carefully dissected and reflected. Great care will be taken to avoid any dura damage, with immediate repair of any incidental dura tears.
- The custom-made Titanium or PEEK implant will then be positioned to fit the defect and securely fixed in place.
- Subcutaneous drainage will be set up following the suturing of the incisions.

In this multicentre study, personnel will undergo centralized training to ensure consistent understanding of the intervention protocols across all centers, achieved through streamlined instructions and ongoing educational sessions. Management of any postoperative complications will be tailored to individual patient needs and will be recorded systematically across all sites to assess the safety and efficacy of the interventions. The medical decisions and surgical procedures are standardized to the extent possible within the non-randomized design to mitigate any influence of trial participation on treatment decisions. This ensures that the study’s findings will provide robust and generalizable data regarding the optimal timing and materials for cranioplasty after decompressive craniectomy.

### 2.5. Outcome measures

The primary outcome is the Barthel Index (BI), which ranges from 0 (worst) to 100 (best), measured at pre-cranioplasty (CP) and 6 months post-CP. The BI is a commonly used scale that measures performance in activities of daily living and is based on 10 variables describing mobility and self-care, reflecting the extent to which patients live independently following discharge [24,25].

In addition to the primary outcome, we will evaluate the following secondary outcomes to provide a comprehensive view of patient recovery and well-being:

1. **Mortality**: Patient survival status will be tracked up to the 6-month follow-up, capturing any deaths that occur post-cranioplasty.
2. **Quality of Life**: Quality of life will be assessed at baseline and follow-up intervals using the EQ-5D, a validated instrument that provides insights into the patient’s general health, mobility, self-care, usual activities, pain/discomfort, and anxiety/depression.
3. **Neurological and Functional Outcomes**:(1)Glasgow Coma Scale (GCS) and Glasgow Outcome Scale (GOS) will be measured at pre-CP and post-CP intervals to indicate long-term functional outcomes and overall morbidity[26]. (2)Mini-Mental State Examination (MMSE) will be administered to assess cognitive function, including orientation, memory, attention, and language abilities, at pre-surgery and 6 months post-CP [27].
4. **Postoperative Complications:** Incidences of complications within 6 months post-CP will be recorded, including postoperative hydrocephalus (requiring cerebrospinal fluid diversion), new-onset seizures, subgaleal effusion, superficial surgical site infections, and implant plate complications. Diagnoses will primarily be confirmed through CT or MRI imaging and clinical assessments.
5. **Hospital Length of Stay**: The total duration of hospital stay following surgery, including time in the intensive care unit and general hospital ward, will be recorded to evaluate the healthcare burden associated with each timing group.

### 2.6. Data collection

Once a patient is identified as meeting the eligibility criteria, they will be enrolled into the study by a local investigator or designated study group member. Each participant will then provide informed consent and receive a unique patient identification number to ensure confidentiality and accurate tracking throughout the study. Data collection will be meticulously organized using a dedicated Case Report Form (CRF) that captures essential demographic, clinical, treatment, and follow-up information.

The key data points to be collected through the CRF include:

1. Demographic and General Information: This includes age, gender, comorbidities, results of physical examinations, and family health history.
2. Clinical Information: Details about the cause and mechanism of the initial decompressive craniectomy, the timing of the procedure, the location and dimensions of the skull defect, and the condition of the skin flap (depressed, level with cranial margins, or bulging). Assessments of neurological functions and cognitive conditions will be conducted using established metrics such as the Barthel Index (BI), Glasgow Coma Scale (GCS), Glasgow Outcome Scale (GOS), and Mini-Mental State Examination (MMSE).
3. Treatment Details: Includes intraoperative observations, postoperative care, and the incidence and management of any complications observed during hospitalization. Routine postoperative CT scans will be performed to monitor for subgaleal effusion and other potential issues.
4. Follow-Up Assessments: These will be carried out in the outpatient clinics at participating sites to continuously assess and record clinical data such as GCS, GOS, MMSE, BI, and any complications that arise after cranioplasty. Follow-up visits are scheduled for 1, 3, and 6 months post-cranioplasty to ensure a comprehensive evaluation over time.

Data from the CRF will be systematically digitized into electronic case report forms according to a predefined schedule. The organization and timing of data collection are outlined in **Fig 1**.

### 2.7. Data management

The entire research database is hosted and managed by West China Hospital. All collected CRF data will be supervised by the Data Management Committee at West China Hospital. Access to patient data will be restricted to approved data management personnel. Only members of the research team and participating site staff will have access to the data. Additionally, Clinical Research Associates (CRAs) at the study sites will ensure that the study processes and research plans are being properly followed.

### 2.8. Sample size and power justification

Our sample size calculation is based on the primary objective of assessing the impact of cranioplasty timing on the 6-month BI scores, adjusted for baseline BI scores, age, gender, skull defect size, and severity of the initial injury. To determine these effects, an Analysis of Covariance (ANCOVA) will be utilized. With a statistical significance level set at 0.05 and a power of 0.8, we aim to detect a small to medium effect size of Cohen’s f of 0.16. Based on these parameters, the calculated sample size required is 380 participants in total. Considering an expected attrition rate of 24%, the total sample size needed is approximately 500 participants. To ensure balanced group sizes, approximately 250 participants will be needed for each group (early and late cranioplasty).

### 2.9. Statistical analysis

The study analysis and reports will be based on the SPIRIT guidelines [28]. This study employs a prospective, multicenter, non-randomized controlled design to evaluate the effects of early versus late cranioplasty on neurological outcomes over multiple time points. The primary and secondary outcomes, including the BI, MMSE, GCS, and GOS, are assessed at baseline, 1 month, 3 months, and 6 months post-cranioplasty. Demographic and clinical characteristics of the participants will be summarized using descriptive statistics, with continuous variables described as means ±  standard deviations or medians with interquartile ranges, and categorical variables reported as frequencies and percentages. To analyze the impact of the timing of cranioplasty on the 6-month BI scores, while controlling for baseline BI scores, an ANCOVA will be conducted. This will specifically allow us to assess the primary hypothesis regarding the effect of early versus late cranioplasty at a fixed follow-up point.

In addition, mixed-effects models will be employed to evaluate changes in outcomes across all time points. These models will include fixed effects for time, treatment group, and their interaction to assess how outcomes evolve over time. To account for hierarchical and clustered data, random intercepts will be incorporated to control for variability at three levels: hospitals, surgeons, and patients. This hierarchical structure allows for patients to be nested within surgeons and surgeons within hospitals, addressing potential variability due to environmental and procedural differences across the 10 hospitals involved in the study.

To reduce selection bias stemming from non-randomized group assignment, we will use propensity scores calculated from baseline characteristics and potential confounding variables that might influence the likelihood of receiving early versus late cranioplasty. These propensity scores will be included as covariates in the models to adjust for imbalances between the groups due to clinical judgment, patient preference, and logistical factors.

Additional confounders, such as age, gender, skull defect size, and initial injury severity, will also be adjusted for in both the ANCOVA and mixed-effects models to isolate the effect of cranioplasty timing. Missing data will be handled using multiple imputation techniques, ensuring a complete-case analysis to maintain statistical power and reduce bias. All statistical tests will be two-sided, with a significance level set at 0.05. Given the multiple outcomes and time points analyzed, adjustments for multiple comparisons, such as the Bonferroni correction, will be considered to control the family-wise error rate.

### 2.10 Ethics and dissemination

This study has received ethical approval from the Ethics Committee and Institutional Review Board of West China Hospital, Sichuan University. The study is sponsored by the 1.3.5 Project for Disciplines of Excellence - Clinical Research Incubation Project at West China Hospital, Sichuan University and National Natural Science Foundation of China. All participating hospital sites must obtain approval from their local ethics committee or comply with the decisions made by our ethics committee.

Prior to enrollment, each participant or their legally authorized representative (if the participant is unable to consent) will provide fully informed, written consent. The consent process will ensure that all participants are thoroughly informed about the study’s aims, procedures, and their right to withdraw from the study at any time without affecting their ongoing medical care. This process underscores our commitment to upholding the highest standards of research ethics and participant rights.

Upon completion of the study, the findings will be shared with the scientific community and the public through presentations at international conferences, publications in peer-reviewed journals, and inclusion in student theses. This broad dissemination strategy is designed to maximize the impact of the study’s findings on clinical practice and future research.

Clinicians who contribute at least one case to the study will be eligible for authorship on any publications that result from the data they helped collect, recognizing their valuable contributions to the research. These measures ensure that the study adheres to stringent ethical standards and that the results are shared widely to advance knowledge in the field of neurosurgery and improve patient care practices globally.

## 4. Discussion

The timing of cranioplasty following decompressive craniectomy remains a debated issue in neurosurgery, with prior research providing mixed results on the relationship between surgical timing, complications, and neurological outcomes [10,29,30]. This study, representing the largest prospective, non-randomized clinical trial in China on the effects of cranioplasty timing, addresses this gap by comparing neurological outcomes and complication rates at different surgical intervals.

In the absence of a universally accepted definition for the optimal timing of cranioplasty, our study adopted a dichotomization at 3 months post-decompression, aligning with international consensus recommendations [16]. This threshold not only facilitates clinical benchmarking but also standardizes research parameters, making findings more comparable across studies. Despite this, the literature suggests that commonly used neurological assessments like the GCS, GOS, and KPS may not adequately capture the nuanced improvements that cranioplasty can foster [18]. In contrast, the BI has been identified as a potentially more sensitive measure for evaluating neurological function and has been used extensively in our study alongside the MMSE to gauge cognitive performance [7].

Given the extensive variability in patient populations and hospital practices across China’s vast healthcare landscape, this study is expected to provide insights into how timing variations might affect the management of cranioplasty. The findings are expected to be instrumental in refining clinical protocols and may influence future guidelines on cranioplasty timing. By shedding light on these important aspects, the study contributes significantly to enhancing patient outcomes in post-decompressive craniectomy care, providing a robust foundation for clinical decision-making concerning the timing of cranioplasty.

In conclusion, as we navigate the complexities of neurosurgical care, this non-randomized trial aims to generate evidence that supports structured approaches to cranioplasty timing, potentially leading to better recovery trajectories for patients suffering from severe brain injuries.

### Strengths and limitations of this study

- This prospective, non-randomized clinical trial is designed to be conducted across multiple clinical centers, aming to include a diverse patient demographic to enhance external validity.
- The study employs multiple standardized indices (Barthel Index, Mortality, Quality of life, Mini-Mental State Examination, Glasgow Coma Scale, Glasgow Outcome Scale) to assess general health, functional recovery, cognitive performance, and complication rates, providing a holistic view of the impacts of cranioplasty timing.
- The dischotomization at 3 months post-decompression for cranioplasty timing is intended to aligh with international consensus recommendations. This facilitates clinical benchmarking and standardizes research parameters, making the findings more comparable across studies.
- With a follow-up period of 6 months, the study may not capture long-term outcomes and complications associated with cranioplasty.

## Supporting information

S1 File SPIRIT checklist. (DOC)

S2 File Research Proposal. (DOCX)

## Abbreviations

ANCOVA: Analysis of Covariance
BI: Barthel Index
CP: Cranioplasty
CRAs: Clinical Research Associates
CRF: Case Report Form
CSF: Cerebrospinal Fluids
CT: Computed Tomography
DC: Decompressive Craniectomy
GCS: Glasgow Coma Scale
GOS: Glasgow Outcome Scale
HA: Hydroxyapatite
MMSE: Mini-Mental State Examination
MRI: Magnetic Resonance Imaging
PEEK: Polyetheretherketone
PMMA: Polymethylmethacrylate
