# Supplementary material for: Comparative effectiveness of different surgical timings on neurological outcomes for cranioplasty: Protocol for a prospective non-randomized controlled trial
Source: PLoS One. 2025 Mar 10;20(3):e0318841. doi: 10.1371/journal.pone.0318841 (PMC11892811; doi:10.1371/journal.pone.0318841)
Supplement: S2 File — (DOCX) [file pone.0318841.s002.docx]

Research Proposal

# Comparative Effectiveness of Different Surgical Timings on Neurological Outcomes for Cranioplasty

# Abstract

## Research Design (Multiple choices allowed)

□ Case-control study
□ Cohort study
□ Cross-sectional study
□ Randomized controlled trial

🗹 non-Randomized controlled trial
□ Blinded
□ Others:

## Total Number of Cases

500

## Risk/Benefit Analysis

Decompressive craniectomy can effectively treat refractory intracranial hypertension that persists despite medical intervention. Postoperatively, the risk of trauma increases due to the absence of a protective skull flap, and the risk of complications such as infection, hydrocephalus, and cerebrospinal fluid leakage also rises. Although studies show that the complication rate after cranioplasty can reach 30%, long-term research indicates that cranioplasty can prevent some complications and aid in neurological recovery and appearance restoration, helping patients return to normal life sooner. Regarding whether the timing of cranioplasty affects the incidence of complications and neurological outcomes, existing evidence presents conflicting views. Despite a recent comprehensive systematic review concluding that early cranioplasty can enhance neurological function, the strength of the supporting evidence is relatively low, including only retrospective and small-scale studies. Therefore, the relationship between the timing of cranioplasty and its outcomes remains controversial.

## Risk Assessment

🗹 Not greater than minimal risk
□ Greater than minimal risk

Minimal risk: The likelihood and magnitude of harm or discomfort anticipated in the research are not greater than those ordinarily encountered in daily life or during the performance of routine physical or psychological examinations.

# I. Research Background

Decompressive craniectomy effectively relieves refractory intracranial hypertension caused by cranial injuries. Secondary cranioplasty is a necessary and common neurosurgical procedure aimed at restoring skull integrity and assisting in the recovery of neurological function. Despite the relative simplicity of cranioplasty, it is associated with a high complication rate (15%-35%). The main issues surrounding cranioplasty include:
1. Management of complications secondary to decompressive craniectomy;
2. Choice of materials for cranioplasty;
3. Interval between decompressive craniectomy and cranioplasty;
4. Management of hydrocephalus following decompressive craniectomy and cranioplasty;
5. Management of temporal muscle atrophy post-cranioplasty.

There are ongoing controversies regarding the choice of materials for cranioplasty, such as traditional autologous bone, titanium, and recent polyetheretherketone materials. The optimal timing for cranioplasty and its impact on postoperative complications is also debated. This study aims to analyze the clinical outcomes of cranioplasty performed at different times and to determine the optimal timing for cranioplasty using a larger sample size to guide clinical treatment.

# II. Research Objectives

## Primary Objectives

1. Analyze the neurological outcomes of cranioplasty performed at different times.

## Secondary Objectives

1. Analyze the incidence of postoperative complications of cranioplasty performed at different times;
2. Establish relevant clinical predictive models.

# III. Research Design Type, Principles, and Sample Sources

## 1. Research Design

### Overall Design

This study is designed as a prospective, multicenter, non-randomized controlled trial to evaluate the outcomes of early versus late cranioplasty in patients following decompressive craniectomy. The trial is organized to compare two distinct patient groups based on the timing of their cranioplasty procedure. The early group will consist of patients receiving cranioplasty within 6 weeks to 3 months post-decompression, while the late group will include those undergoing the procedure later than 3 months post-decompression. The trial will enroll participants from 10 clinical centers across China, selected based on their high standards of care and previous experience with similar studies. As postoperative neurological evaluation is the primary indicator, the main objective is to analyze neurological outcomes of cranioplasty performed at different times. All analyses will be conducted anonymously to protect patient privacy.





### Sample Size

Recruitment for this study is scheduled to commence in January 2025 and is anticipated to conclude by January 2026. We aim to enroll a minimum of 500 patients who have undergone decompressive craniectomy. The study will run until August 2026, which marks the final follow-up assessments for the last recruited participants.

## 2. Medical Records / Specimen Names, Sources, Periods, Acquisition, Processing, and Disposal

Patients undergoing cranioplasty in our department from 2025 to 2026 due to various reasons leading to decompressive craniectomy will be prospectively collected and analyzed. All analyses will be conducted anonymously, protecting patient privacy and data confidentiality.

# IV. Quality Control and Assurance

## 1. Subject Compliance

Patient follow-up management is key to ensuring research quality. A standardized follow-up plan will be developed. Patients will be informed and educated at the start of the study. Follow-up visits will be scheduled at 3 months and 6 months post-cranioplasty until follow-up data collection is completed. The study will terminate once 6-month follow-up data is collected.

## 2. Researcher Training

Researchers involved in patient follow-up will receive systematic clinical research training before the study begins. A comprehensive follow-up reminder system will be established:
1. Follow-up cards will be designed and distributed to remind patients of follow-up visits, or researchers responsible for follow-up will remind patients to visit the hospital;
2. The follow-up interval may be appropriately extended, such as conducting complete follow-up examinations every 3 or 6 months.

## 3. Data Collection and Management

Follow-up will primarily be conducted in outpatient clinics, with information recorded in case report forms and entered into an electronic data collection database. The electronic database will be developed and maintained by researchers independent of West China Hospital, Sichuan University. Data will be organized and analyzed by personnel with a background in epidemiology. Considering potential biases and confounding factors in real-world research, measures will be taken to include these factors in the research plan and case report forms. Statistical methods such as stratified analysis, multivariate analysis, and propensity score will be used to control and correct these factors during data analysis.

# V. Ethical Principles and Requirements

This clinical research will adhere to the Declaration of Helsinki and relevant regulations of the National Health and Family Planning Commission of the People's Republic of China. As a prospective observational study, it involves no intervention, only prospective data collection. Personal information of subjects will be anonymized, ensuring no risk to subjects and no adverse effects on their rights and health. Research data will be stored at West China Hospital, Sichuan University. Researchers, regulatory authorities, and the ethics review committee may review the data. Public reports will not disclose the personal identity of subjects. Efforts will be made to protect the privacy and personal information of subjects within the legal scope.

# VI. Research Progress

• October 2023 - December 2024: Research preparation phase;
• June 2025 - June 2026: Researchers will collect and cross-verify patient diagnosis and treatment information;
• June 2026 - December 2026: Follow-up of patients to assess changes in functional outcomes and complications;
• May 2027: Organize follow-up results, perform statistical analysis, and write research articles.

# VII. Participants

| Name | Title | Specialty | Task | GCP Training Certificate |
| --- | --- | --- | --- | --- |
| Junwen Guan | Chief Physician | Neurosurgery | Experimental Design | ✓ |
| Jingguo Yang | None | Neurosurgery | Experimental Design, Data Collection, Statistics, Article Writing | ✓ |
| Xiaoyu Yang | None | Neurosurgery | Data Collection | - |
| Junjie Wang | None | Neurosurgery | Data Collection | - |
| Ke Ju | Assistant Researcher | School of Public Health | Data Quality Control and Analysis | ✓ |

# Main References

1. Shepetovsky D et al. Complications of cranioplasty in relationship to traumatic brain injury: a systematic review and meta-analysis. Neurosurg Rev. 2021 Mar 8. doi: 10.1007/s10143-021-01511-7.

2. Zhang Q et al. A Large Multicenter Retrospective Research on Embedded Cranioplasty and Covered Cranioplasty. World Neurosurg. 2018 Apr;112:e645-e651. doi: 10.1016/j.wneu.2018.01.114.

3. Shih FY et al. Risk factors for seizures after cranioplasty. Seizure. 2019;66:15-21. doi: 10.1016/j.seizure.2018.12.016.

4. Morton RP et al. Timing of cranioplasty: a 10.75-year single-center analysis of 754 patients. J Neurosurg.2018 Jun;128(6):1648-1652.

5. Honeybul S, Ho KM. Long-term complications of decompressive craniectomy for head injury. J Neurotrauma. 2011 Jun;28(6):929-35. doi: 10.1089/neu.2010.1612.

6. Chaturvedi J et al. Complications of cranioplasty after decompressive craniectomy for traumatic brain injury. Br J Neurosurg. 2016;30(2):264-8.

7. Iaccarino C et al. Consensus statement from the international consensus meeting on post-traumatic cranioplasty. Acta Neurochir (Wien). 2021 Feb;163(2):423-440.

8. Lilja-Cyron A et al. Long-Term Effect of Decompressive Craniectomy on Intracranial Pressure and Possible Implications for Intracranial Fluid Movements. Neurosurgery. 2020 Feb 1;86(2):231-240. doi: 10.1093/neuros/nyz049.

9. Lilja-Cyron A et al. Intracranial pressure before and after cranioplasty: insights into intracranial physiology. J Neurosurg. 2019 Oct 18:1-11.
